# The epilepsy-associated protein TBC1D24 is required for normal development, survival and vesicle trafficking in mammalian neurons

**DOI:** 10.1093/hmg/ddy370

**Published:** 2018-10-17

**Authors:** Mattéa J Finelli, Davide Aprile, Enrico Castroflorio, Alexander Jeans, Matteo Moschetta, Lauren Chessum, Matteo T Degiacomi, Julia Grasegger, Alexis Lupien-Meilleur, Andrew Bassett, Elsa Rossignol, Philippe M Campeau, Michael R Bowl, Fabio Benfenati, Anna Fassio, Peter L Oliver

**Affiliations:** 1 Department of Physiology, Anatomy and Genetics, University of Oxford, Parks Road, Oxford, UK; 2 Department of Experimental Medicine, University of Genoa, Genoa, Italy; 3 Center of Synaptic Neuroscience and Technology, Istituto Italiano di Tecnologia, Largo Rosanna Benzi 9, Genoa, Italy; 4 Department of Pharmacology, University of Oxford, Mansfield Road, Oxford, UK; 5 MRC Harwell Institute, Harwell, Oxfordshire, UK; 6 Department of Chemistry, Durham University, South Road, Durham, UK; 7 CHU Ste-Justine, Departments of Neurosciences and Pediatrics, Université de Montréal, Montreal, QC, Canada; 8 Cellular Operations, Wellcome Sanger Institute, Wellcome Genome Campus, Hinxton, Cambridgeshire, UK; 9 IRCCS Ospedale Policlinico San Martino, Largo Rosanna Benzi 10, Genoa, Italy

## Abstract

Mutations in the Tre2/Bub2/Cdc16 (TBC)1 domain family member 24 (*TBC1D24*) gene are associated with a range of inherited neurological disorders, from drug-refractory lethal epileptic encephalopathy and DOORS syndrome (deafness, onychodystrophy, osteodystrophy, mental retardation, seizures) to non-syndromic hearing loss. TBC1D24 has been implicated in neuronal transmission and maturation, although the molecular function of the gene and the cause of the apparently complex disease spectrum remain unclear. Importantly, heterozygous *TBC1D24* mutation carriers have also been reported with seizures, suggesting that haploinsufficiency for TBC1D24 is significant clinically. Here we have systematically investigated an allelic series of disease-associated mutations in neurons alongside a new mouse model to investigate the consequences of *TBC1D24* haploinsufficiency to mammalian neurodevelopment and synaptic physiology. The cellular studies reveal that disease-causing mutations that disrupt either of the conserved protein domains in TBC1D24 are implicated in neuronal development and survival and are likely acting as loss-of-function alleles. We then further investigated TBC1D24 haploinsufficiency *in vivo* and demonstrate that TBC1D24 is also crucial for normal presynaptic function: genetic disruption of *Tbc1d24* expression in the mouse leads to an impairment of endocytosis and an enlarged endosomal compartment in neurons with a decrease in spontaneous neurotransmission. These data reveal the essential role for TBC1D24 at the mammalian synapse and help to define common synaptic mechanisms that could underlie the varied effects of *TBC1D24* mutations in neurological disease.

## Introduction

The regulation of synaptic transmission is a key pathological mechanism in disease, with mutations in genes that mediate the synaptic vesicle (SV) cycle joining an ever-expanding group of neurological disorders recently termed as the synaptopathies (1,2). These disorders include inherited forms of epilepsy caused by presynaptic proteins such as MUNC18–1 [encoded by syntaxin-binding protein 1 (STXBP1)] (3,4), Synapsin I (SYN1) (5,6), syntaxin 1B (STX1B) (7,8) and synaptosome-associated protein 25B (SNAP25B) (9), and there are clues from studies in model organisms that many more SV-associated genes will play a central role in diseases characterized by seizures and/or neurodevelopmental delay (10,11).

One of the more recently discovered causes of familial epilepsy is the Tre2/Bub2/Cdc16 (TBC)1 domain family member 24 (*TBC1D24*) gene; over 50 mutations are now associated with a range of inherited neurological disorders, including myoclonic epilepsy, epileptic encephalopathy and DOORS syndrome (deafness, onychodystrophy, osteodystrophy, mental retardation and seizures) (12,13). Additional clinical features reported include neurodevelopmental delay, neurodegeneration and non-syndromic hearing loss (12). However, despite the obvious pleiotropic effects of *TBC1D24* mutations, no clear phenotype–genotype correlation is apparent to date (13).

Initial functional studies demonstrated that the *Drosophila* TBC1D24 orthologue, *skywalker (sky)*, regulates the size and content of endosome-dependent SV pools at the larval neuromuscular junction (NMJ), with loss of *sky* leading to increased neurotransmission (14,15). Most recently, a cationic phosphoinositide binding pocket was identified in the *sky* N-terminal TBC domain and the accumulation of endosomal vesicles alongside seizure-related phenotypes were observed in flies expressing certain DOORS-associated mutations at positions that were conserved in *Drosophila* (16). In rodent systems, acute knockdown of *Tbc1d24* perturbs the migration and dendritic arborization of cortical pyramidal neurons, while conversely, overexpression of *Tbc1d24* induces neurite outgrowth and differentiation; both these particular functions were suggested to be mediated by the TBC domain acting as a modulator of Rab-GTPase activity (13,17–19). In addition, TBC1D24 has been implicated in oxidative stress resistance and cell survival via the highly conserved C-terminal TLDc domain that is shared with a family of proteins including oxidation resistance 1 (OXR1) and nuclear receptor co-activator 7 (NCOA7) (20,21). Yet, despite these data, the molecular function of TBC1D24 is still unclear, particularly when considering the distinct roles of the two conserved protein domains and the potential for allosteric interactions between them. Furthermore, the effects of TBC1D24 disruption on synapse biology is yet to be studied in a mammalian genetic system.

**Figure 1:**
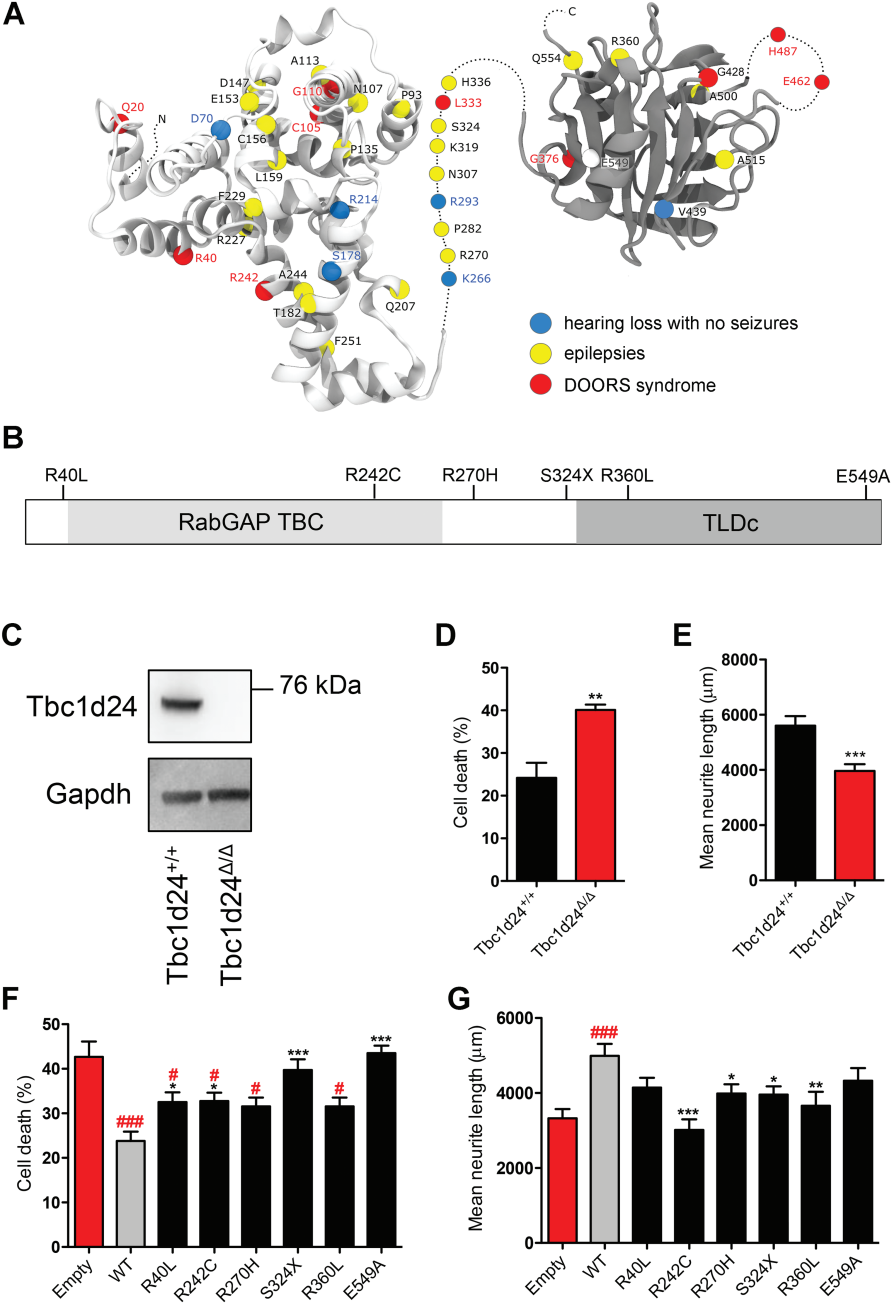
Disease-causing mutations in *TBC1D24* influence neuronal cell differentiation and sensitivity to oxidative stress. **(A)** Three-dimensional structural model of TBC1D24 indicating the positions of published pathogenic mutations classified into three general disease classes (yellow, red, blue) as shown (12,13). E549, the most conserved residue of the TLDc domain is also shown (white). **(B)** The TBC1D24 mutations investigated in this study include those situated just before (R40L) and within the TBC (R242C) domain and in the TLDc domain (R360L). S324X represents a frame-shift mutation (S324Tfs^*^3) that results in a premature stop codon (12,13). The E549A mutation has been described previously as disrupting the function of TBC1D24 (20). **(C)** Western blot demonstrated a complete loss of TBC1D24 protein expression in CRISPR-deleted N2a cells (*Tbc1d24^Δ/Δ^*). GAPDH was used as a loading control. **(D**,**E)** Untransfected *Tbc1d24^Δ/Δ^* cells were more sensitive to arsenite treatment than control N2a cells (D) and grew shorter neurites (*N* > 100 neurites from 3 independent experiments) (E). **(F**,**G)***Tbc1d24^Δ/Δ^* cells transfected with TBC1D24 mutants presented an increased sensitivity to arsenite treatment compared to *Tbc1d24^Δ/Δ^* cells transfected with WT TBC1D24 (F) and grew shorter neurites (G), similar to cells transfected with an empty control vector (*N* > 70 neurites). All data are expressed as mean ± SEM. (F,G) ^*^P < 0.05; ^**^P < 0.01; ^***^P < 0.001; ANOVA followed by Dunnett’s multiple comparison test compared to TBC1D24 WT vector. ^#^*P* < 0.05; ^##^*P* < 0.01; ^###^*P* < 0.001; ANOVA followed by Dunnett’s multiple comparison test compared to empty vector. (D,E) Unpaired *t*-test.

The predominance of recessive and compound heterozygous disease mutations in *TBC1D24* - often with premature termination mutations leading to nonsense-mediated decay in some cases - suggests a loss-of-function mechanism, and this is supported by the significant reduction of TBC1D24 protein expression in patient cells (22,23). Importantly, individuals with heterozygous *TBC1D24* mutations - often relatives of more severely affected individuals - have also been reported with seizures, suggesting that haploinsufficiency for *TBC1D24* can be detrimental (13,24–27). Further evidence for the significance of *TBC1D24* haploinsufficiency is provided by the very recent description of heterozygous microdeletions spanning *TBC1D24* and a small number of adjacent genes; these individuals display epilepsy, microcephaly and developmental delay (28).

How such mutations influence the multiple functions of this gene in neuronal cell survival and synaptic function is unknown. Therefore, here we have systematically analysed disease-associated mutations in neurons combined with the study of synaptic physiology in the first mouse model of *TBC1D24* haploinsufficiency; our data demonstrate that the TBC and TLDc domains are both functionally implicated in neuronal development and survival and that TBC1D24 is essential for normal presynaptic function *in vivo.*

## Results

TBC1D24 is unique in a sense that it contains both a TBC and TLDc domain, yet the functional significance of this architecture in relation to disease-causing mutations is not understood. We mapped TBC1D24 mutations onto a new structural prediction of the human TBC and TLDc domains, suggesting that there is no obvious clustering of a certain clinical sub-type or severity (Fig. 1A). To quantify this assumption, the distance between disease-associated amino acids was calculated based on a new model of the TBC domain, and no disease appears to be associated to mutations in a specific protein region (Supplementary Material, Fig. S1). Therefore, we next utilized a systematic experimental approach to study the functional impact of a series of disease mutations on two key cellular parameters known to be influenced by TBC1D24, oxidative stress resistance (20) and neurite outgrowth (17,18). As the functional data available in mammalian systems to date have been limited to acute embryonic knockdown or overexpression studies in wild-type (WT) rodent tissues or cells, we chose to generate a CRISPR-Cas9-mediated TBC1D24 knockout neuronal cell line (*Tbc1d24^Δ/Δ^*) and studied the expression of an allelic series TBC1D24 mutants compared to WT TBC1D24. Mutants were selected to represent a broad clinical spectrum of recessively inherited mutations, as well as spanning the full length of the protein including both conserved domains (Fig. 1B). Mutants represented DOORS syndrome (R40L and R242C), infantile myoclonic epilepsy (R270H), early-onset epileptic encephalopathy (S324X) and progressive myoclonic epilepsy with neurodegeneration (R360L) (12,13). Hence, this approach not only avoids the confounding, compensatory effects of endogenous WT TBC1D24 present in the system, but also allows us to assess potential correlations between genotype and phenotype across two key functional parameters.

We first confirmed that the *Tbc1d24^Δ/Δ^* Neuro2a (N2a) cell line lacked any TBC1D24 at the protein level (Fig. 1C). Importantly, these cells were not only viable, but they also demonstrated reduced survival under oxidative stress (Fig. 1D) and grew shorter neurites when differentiated compared to control cells (Fig. 1E). Thus, this cell line facilitates comparative studies of phenotypic rescue by exogenous expression of WT TBC1D24 versus disease mutants. Firstly, for oxidative stress resistance, expression of WT TBC1D24 in the *Tbc1d24^Δ/Δ^* line was able to significantly prevent cell death versus an empty negative control vector (Fig. 1F). As an additional control for this assay, expression of TBC1D24 with a mutation in a highly conserved amino acid proven to abolish the protective function of the TLDc domain was tested (E549A) (20); as expected, this mutation prevented any protection against oxidative stress-induced cell death (Fig. 1F). For the disease-causing mutations, expression of the TBC1D24 truncation mutant (S324X) also conferred virtually no protection against cell death compared to WT TBC1D24 (Fig. 1F). All of the remaining mutants resulted in significantly higher cellular survival compared to the empty vector control, yet none were as protective as expression of the WT protein (Fig. 1F). Secondly, for the analysis of neuronal differentiation, WT TBC1D24 was able to induce neurite outgrowth successfully in *Tbc1d24^Δ/Δ^* cells (Fig. 1G). All of the TBC1D24 mutants tested resulted in some reduction in the efficiency of outgrowth induction, with expression of four of the disease-causing mutations leading to significantly shorter neurite length than WT TBC1D24 expression (Fig. 1G). We also confirmed that these findings were not likely due to variable expression of the exogenous WT and mutant TBC1D24 proteins in N2a cells (Supplementary Material, Fig. S2). Taken together, these data demonstrate that in the absence of WT TBC1D24, expression of all the mutations tested here lead to at least a partial loss-of-function of TBC1D24, both in terms of oxidative stress resistance and neurite outgrowth. Furthermore, the influence of mutations on these key cellular parameters is not confined to a particular region or domain of the TBC1D24 protein sequence.

**Figure 2:**
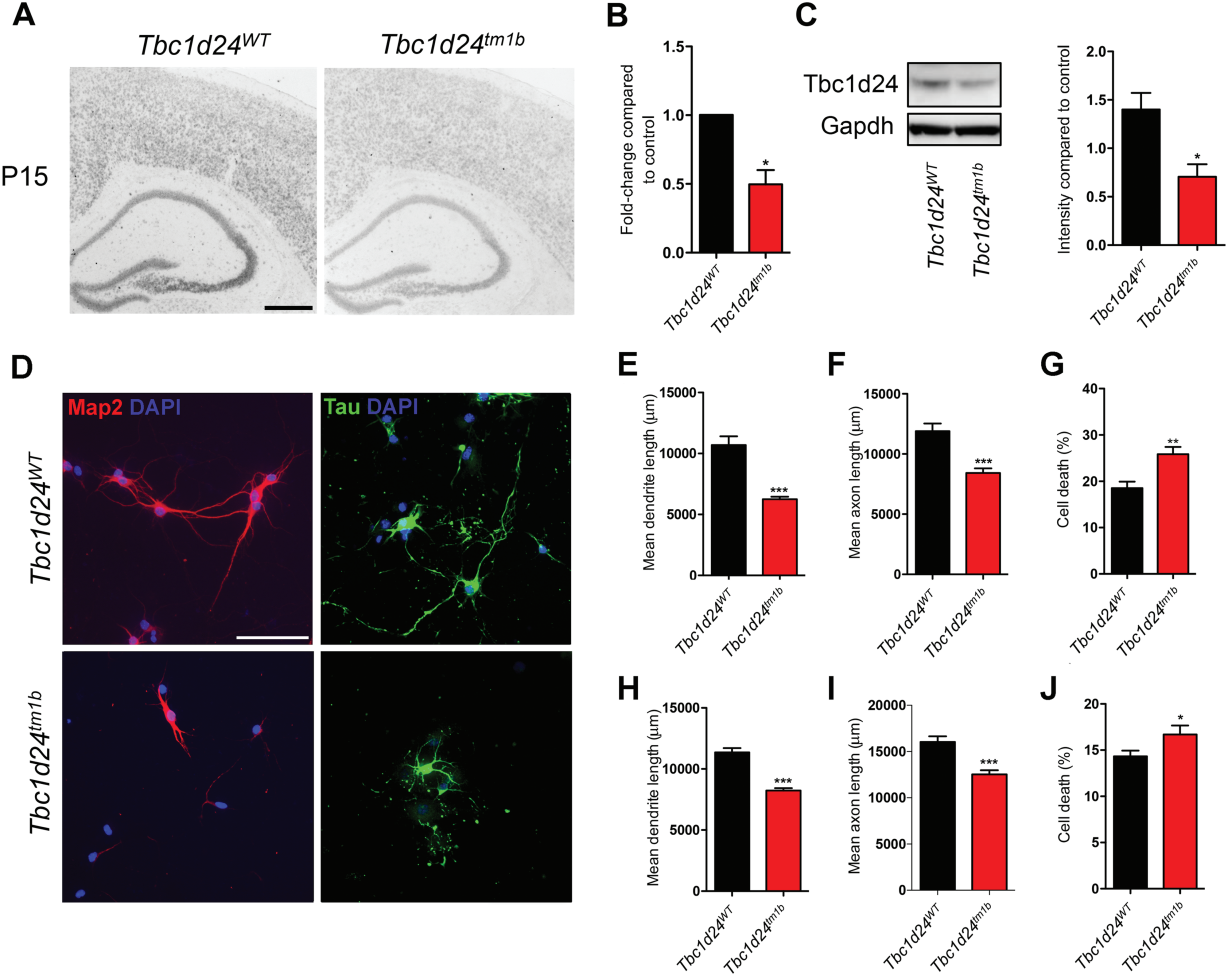
Genetically reduced levels of *Tbc1d24* expression alters primary neuronal maturation and survival. **(A)***In situ* hybridization demonstrating a reduction in *Tbc1d24* expression in the postnatal (P15) mouse brain from *Tbc1d24^tm1b^* mice versus littermate *Tbc1d24^WT^* controls. Scale bar: 0.5 mm. A significant reduction in expression in the hippocampus at P15 is confirmed by qRT-PCR (*N* = 3 animals of each genotype) **(B)** and western blot (*N* = 5 animals of each genotype) **(C);** GAPDH was used as a loading control. **(D)** Representative immunostaining of primary hippocampal cultures from *Tbc1d24^WT^* or *Tbc1d24^tm1b^* with dendritic (Map2) and axonal (Tau) markers. Scale bar: 100 µM. **(E**,**F)** Quantification of dendrite (E) and axonal (F) length in hippocampal neurons (*N* > 100 axons/dendrites from 3 independent preparations of each genotype). **(G)** In the same cultures, the number of apoptotic cells was quantified by cleaved caspase-3 immunostaining after arsenite treatment to induce oxidative stress. **(H**,**I)** Quantification of dendrite (H) and axonal (I) length in primary cortical (*N* > 100 axons/dendrites from 4 independent preparations of each genotype). (**J**) In the same cultures the number of apoptotic cells was quantified as in (G). Data are expressed as the mean ± SEM. (B, C, E–J) ^*^P < 0.05; ^**^P < 0.05; ^***^P < 0.001 unpaired *t*-test compared to *Tbc1d24^WT^*.

To investigate further the consequences of TBC1D24 loss-of-function and haploinsufficiency *in vivo*, we chose to use a viable mammalian genetic system as none have been described to date. Therefore, we utilized a mouse model harbouring one copy of a disrupted Tbc1d24 allele (*Tbc1d24^tm1b^*). These mice express approximately half the level of TBC1D24 compared to WT controls (*Tbc1d24^WT^*) at both the RNA and protein level (Fig. 2A–C). Considering that Tbc1d24 has been implicated previously in neuronal migration, and that human TBC1D24 mutations can cause neurodevelopmental delay, we assessed initially the early postnatal period in *Tbc1d24^tm1b^* mice. The gross structure of the brain appeared normal in *Tbc1d24^tm1b^* animals, and quantification of layer markers in the somatosensory cortex and cell numbers in the hippocampus did not indicate any migration defects or neurodegeneration in mutants compared to *Tbc1d24^WT^* mice (Supplementary Material, Fig. S3). In addition, assessment across the neonatal period showed that *Tbc1d24^tm1b^* mice showed normal growth patterns, and behavioural analyses demonstrated that mutants reached key neurodevelopmental milestones at the same time as controls (Supplementary Material, Fig. S4).

One of the recurrent clinical features of individuals with TBC1D24 mutations is deafness; therefore, we also analysed auditory function and the ultrastructure of the inner ear in *Tbc1d24^tm1b^* mice. First, auditory brainstem response (ABR) recordings were carried out and no differences in auditory threshold were detected between genotypes (Supplementary Material, Fig. S5A). Considering that TBC1D24 is reported to be expressed specifically in the inner hair cells of the cochlea in neonatal mice (29), we next analysed the position and structure of these stereocilia by scanning electron microscopy (SEM) and these data indicated that there was no disorganization or loss of the cochlea hair cell bundles in *Tbc1d24^tm1b^* mice (Supplementary Material, Fig. S5B–E). Thus, combined with the normal ABR thresholds observed, our findings suggest that there is no auditory dysfunction in *Tbc1d24^tm1b^* mutants.

Our *in vitro* data demonstrate that TBC1D24 has an important influence on cellular survival and differentiation. Therefore, to determine the potential significance of *Tbc1d24* haploinsufficiency to neuronal development and response to oxidative stress, we next analysed the properties of primary neurons from *Tbc1d24^tm1b^* mice. Hippocampal and cortical neurons from *Tbc1d24^tm1b^* mutants were analysed at 5 days *in vitro* (DIV) and showed significantly reduced dendritic and axonal growth compared to WT controls (Fig. 2D–F, H, I). Furthermore, primary neurons from both the hippocampus and cortex of *Tbc1d24^tm1b^* mice displayed an increased sensitivity to oxidative stress-induced cell death compared to WT controls (Fig. 2G, J). Therefore, despite generally normal neurodevelopment in mutant mice, the genetic disruption of *Tbc1d24* has a clear impact on the maturation and oxidative stress resistance of neurons.

**Figure 3:**
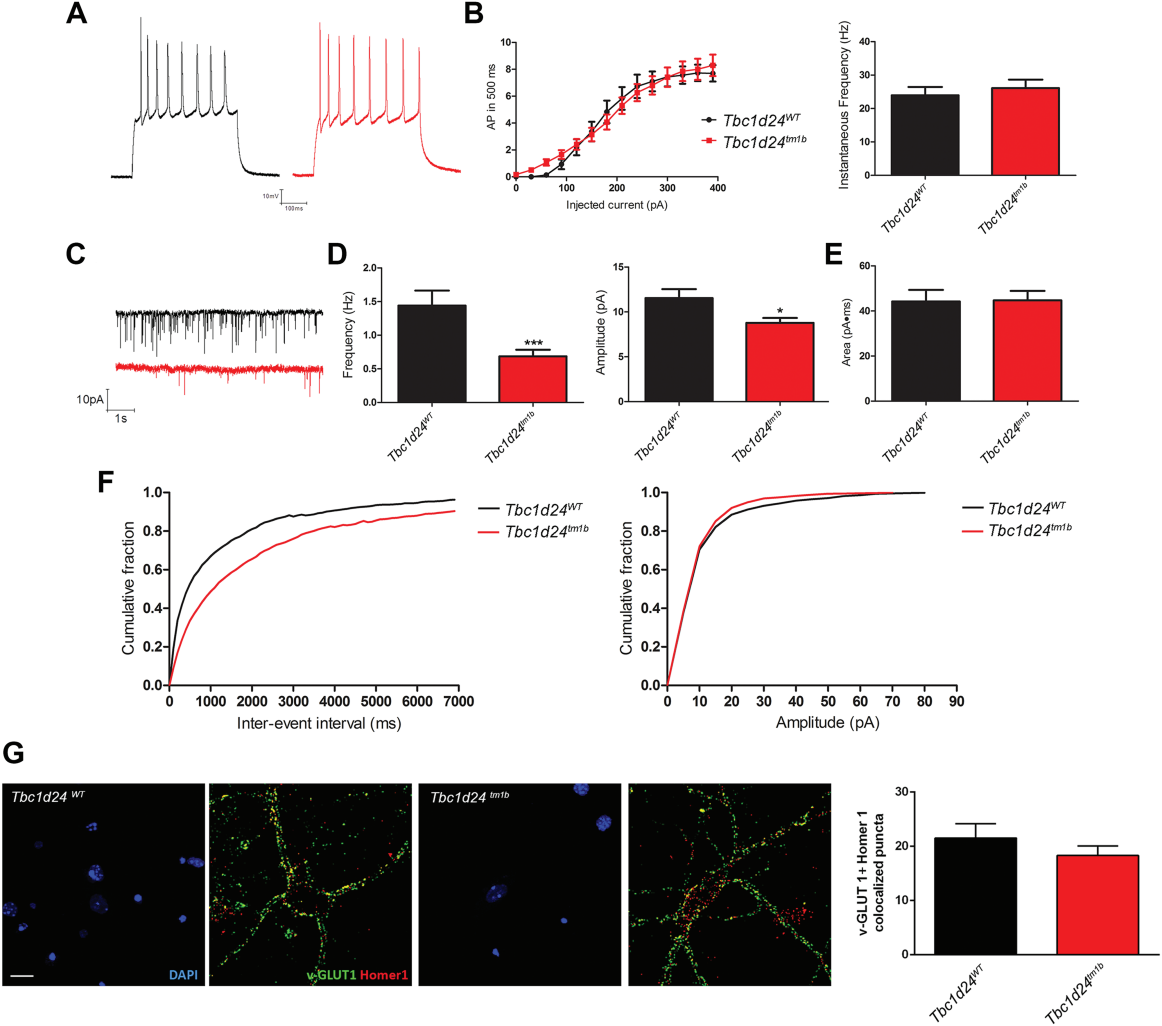
Genetic disruption of *Tbc1d24* expression results in decreased mEPSC frequency with no effect on excitatory synapse number in hippocampal primary neurons. **(A)** Representative current-clamp recordings of spike trains evoked by somatic current injection of 300 pA for 500 ms from hippocampal WT *Tbc1d24^WT^* (black) and mutant *Tbc1d24^tm1b^* (red) mice. **(B)** AP in 500 ms (or mean firing frequency in Hz) versus injected current (pA; left) and averaged instantaneous frequency at 300 pA (right) (*N* = 31 neurons from 4 independent preparations). **(C)** Representative traces of mEPSCs recorded at −70 mV in 14 DIV hippocampal neurons from *Tbc1d24^WT^* (black) and *Tbc1d24^tm1b^* (red) mice. **(D)** Histograms showing average peak frequency and amplitude of mEPSCs. **(E)** Average amplitude of mEPSCs calculated as peak area. **(F)** Cumulative plot of peak frequency (left) and amplitude (right) showing a selective defect in the peak frequency (*N* = 25 and 27 neurons from *Tbc1d24^WT^* and *Tbc1d24^tm1b^* mice respectively from 4 independent preparations). **(G)** Representative images of *Tbc1d24^WT^* and *Tbc1d24^tm1b^* hippocampal culture immunolabelled with the glutamatergic presynaptic and postsynaptic markers v-GLUT1 and Homer1. Nuclei are stained with DAPI. Number of v-GLUT1 and Homer1 positive puncta are automatically counted and normalized to the number of cells in the field (*N* = 28–24 fields per experimental condition from 3 independent preparations). Scale bar: 20 µM. All data are expressed as the mean ± SEM. (B, D, E, G) ^*^P < 0.05; ^***^P < 0.001; unpaired *t*-test.

**Figure 4:**
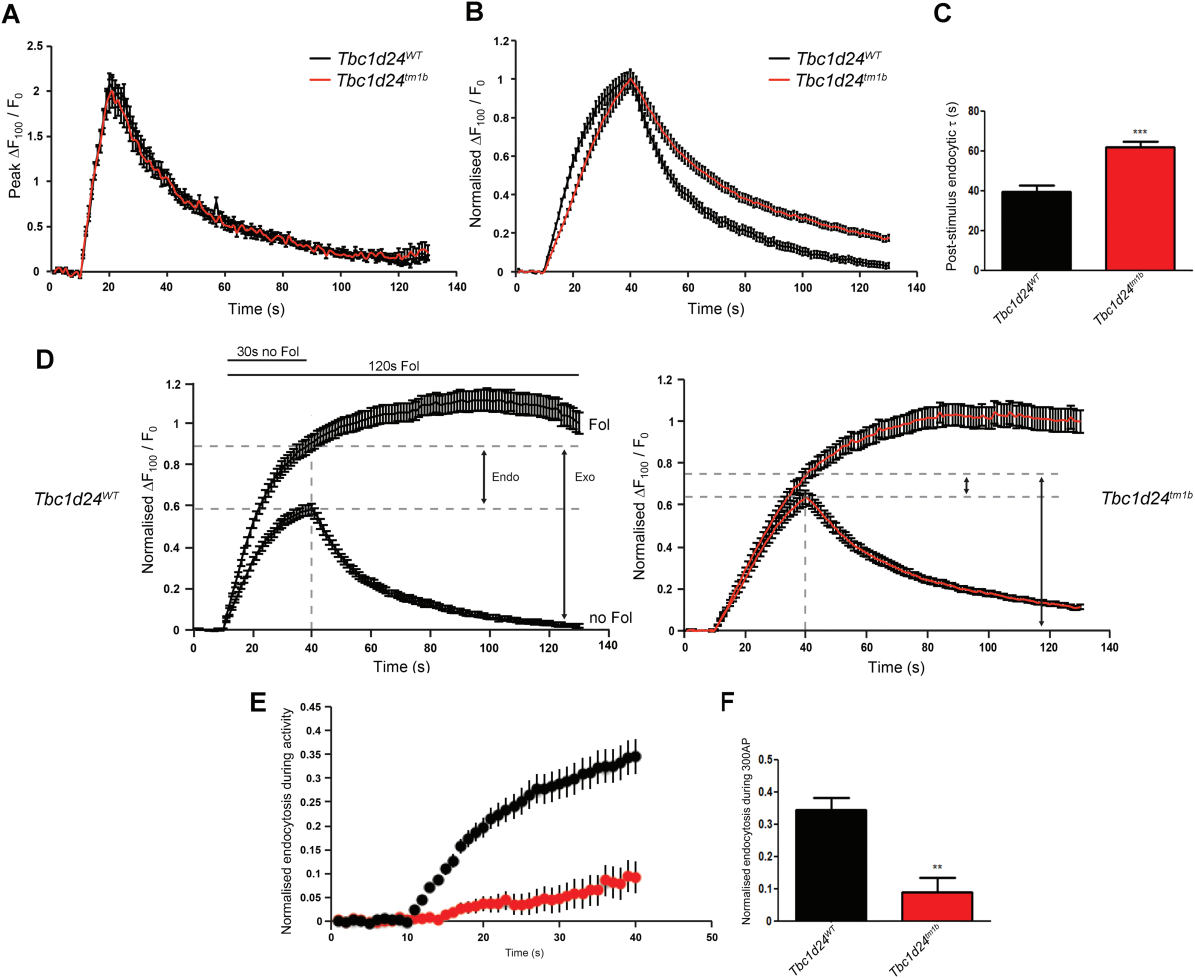
Genetic disruption of *Tbc1d24* expression results in defective endocytosis in primary neurons. **(A)** Average fluorescence traces of hippocampal neurons at 15 DIV expressing the pHluorin SypH 2× in response to 100 stimuli at 10 Hz [*N* = 188 boutons from 8 coverslips (*Tbc1d24^WT^*) or 87 boutons from 5 coverslips (*Tbc1d24^tm1b^*)] indicating no difference in the post-stimulus endocytic time constant. **(B)** Normalized average traces of neurons expressing SypH 2x in response to 300 stimuli at 10 Hz. **(C)** Comparison of average post-stimulus endocytic time constants following the 300 AP stimulus. The decay phases of ΔF traces were fitted with single exponential functions before normalization and the time constants were calculated from the fits [*N* = 113 boutons from 5 coverslips (*Tbc1d24^WT^*) or 190 boutons from 6 coverslips (*Tbc1d24^tm1b^*)]. **(D)** Time course and extent of endocytosis during neuronal activity were measured using folimycin (Fol). Neurons expressing SypH 2× were stimulated at 10 Hz for 30 s in the absence of Fol. After a 10 min rest, neurons were stimulated at 10 Hz for 120 s in the presence of Fol. Images were acquired during each phase of stimulation. Graph shows average SypH 2× traces from *Tbc1d24^WT^* neurons in response to each stimulus. All values were normalized to the maximum fluorescence change at the end of 1200 stimuli in presence of Fol. The short and long vertical arrows indicate the extent of endocytosis and exocytosis at the end of the 300 stimuli (vertical dashed line) at 10 Hz, respectively (*N* = 162 boutons from 5 coverslips). The same experiment was repeated in *Tbc1d24^tm1b^* neurons (*N* = 200 boutons from 6 coverslips). **(E)** Time courses of during-stimulus endocytosis for *Tbc1d24^WT^* and *Tbc1d24^tm1b^* neurons during stimulation (30 s, 10 Hz). Each endocytosis time-course was calculated by subtracting the SypH 2× trace that was acquired in the absence of Fol, from the Fol trace in (D). These traces were fitted with linear functions, the slope of which corresponds to the endocytic rate in arbitrary units per second. **(F)** Average magnitude of endocytosis calculated during delivery of 300 pulses. Data are expressed as the mean ± SEM. (C,F) ^**^P < 0.01; ^***^P < 0.001; unpaired *t*-test.

**Figure 5:**
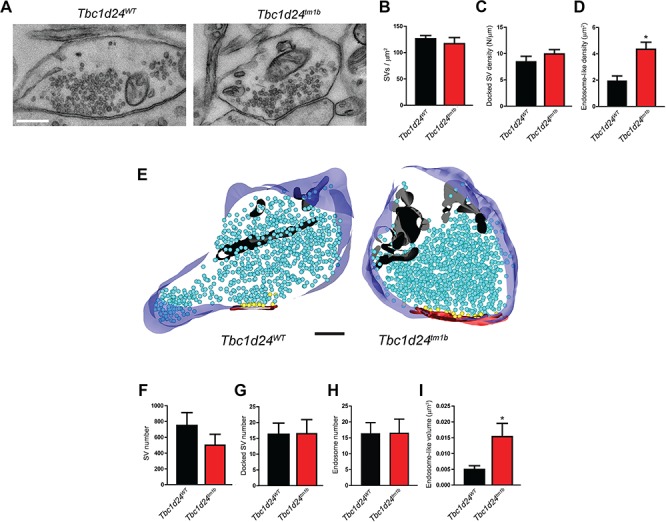
Genetic disruption of *Tbc1d24* expression results in increased endosomal volume in primary neurons. (**A**) Representative TEM images of synapses of cultured cortical neurons from WT *Tbc1d24^WT^* and mutant *Tbc1d24^tm1b^* mice, fixed at 17 DIV. Scale bar: 200 nm. **(B**–**D)** Conventional TEM analysis of nerve terminals from *Tbc1d24^tm1b^* (red bars) revealed a preserved density of both total (H) and docked (I) SVs with respect to control neurons (black bars), with a significant increase in the density of endosomal-like structures. **(E)** Three-dimensional reconstructions of synaptic terminals from serial ultrathin sections; shown are representative three-dimensional reconstructions from 60 nm-thick serial sections obtained WT *Tbc1d24^WT^* and mutant *Tbc1d24^tm1b^* synapses. Total SVs and SVs physically docked at the AZ are depicted as blue and yellow spheres, respectively. The AZ and endosomes are shown in red and black, respectively. Scale bar, 200 nm. **(F**–**H)** Ultrastructural morphometric analysis of three-dimensionally reconstructed synapses displayed similar amount of both total (F) and docked (G) SVs and endosomal number (H). **(I)** The endosomal-like volume was calculated from each 2D analysis of synaptic profile and is expressed as mean ± SEM area of endosomes per μm^3^. Nerve terminal areas and AZ lengths were similar in the two experimental groups (*N* = 105 and 126 synapses for *Tbc1d24^WT^* and *Tbc1d24^tm1b^*, respectively, from three independent preparations). Data are expressed as the mean ± SEM. (D, I) ^*^P < 0.05; Mann–Whitney test.

Studies in the fly suggest that disruption of the TBC1D24 orthologue, *sky*, influences aspects of neurotransmission at NMJs (14), yet very little is known about the role of TBC1D24 in mammalian synaptic physiology that might explain the range of clinical outcomes observed in patients. Therefore, we next analysed primary neurons from *Tbc1d24^tm1b^* mice in detail to determine whether haploinsufficiency for the gene would cause abnormalities in synaptic structure and function. First, the intrinsic excitability of *Tbc1d24^tm1b^* and *Tbc1d24^WT^* hippocampal excitatory neurons was measured at 14 DIV by current-clamp recordings while depolarizing the membrane potential with subsequent current steps (Fig. 3A, B). Both genotypes generated very similar spike trains, with no significant difference in both action potential (AP) rates versus injected current and average instantaneous frequency (Fig. 3A, B). Next, we tested whether excitatory neurotransmission was affected in *Tbc1d24^tm1b^* hippocampal neurons. Miniature excitatory post-synaptic currents (mEPSCs) were continuously recorded at the soma of voltage-clamped neurons held at −70 mV (Vh) in the presence of tetrodotoxin (TTX) to block spontaneous APs, and the mEPSC frequency fell almost 3-fold in *Tbc1d24^tm1b^* compared to *Tbc1d24^WT^* neurons (Fig. 3C). Although this effect was accompanied by a slight but significant decrease in the mean peak current amplitude, the synaptic charge was not altered (Fig. 3D, E). Cumulative analysis confirmed the selective defect in mEPSC frequency in *Tbc1d24^tm1b^* neurons, with no marked effects on the distribution of the current amplitude (Fig. 3F). In principle, these findings could be attributable to either abnormal neuronal maturation resulting in a reduced density of synaptic contacts or abnormal release properties. To test the former hypothesis, the number of synaptic boutons was quantified in parallel hippocampal cultures by co-staining with the presynaptic marker v-GLUT1 and the post-synaptic marker Homer1, and no difference in synaptic density was observed between genotypes (Fig. 3G). Together, these data suggest that TBC1D24 is required to maintain the normal function of excitatory nerve terminals.

Given these findings, alongside the consistent presence of TBC1D24 at the mammalian synapse (30,31), we went on to investigate the presynaptic role of TBC1D24. Initially, we transfected primary hippocampal neurons at the same developmental stages used for patch clamp recordings with a synapto-pHluorin (SypH2x) probe to dissect the significance of TBC1D24 to the exo-endocytotic cycle of SVs. First, stimulation with 100 APs at 10 Hz was carried out as an initial measure of basal exocytosis of the readily releasable pool (RRP). No difference in the mean peak fluorescence and in the time constant of post-stimulus endocytosis was observed between *Tbc1d24^tm1b^ and Tbc1d24^WT^* cells, suggesting no defect in the extent and kinetics of exo-endocytosis following a short stimulus (Fig. 4A). Next, a longer stimulation (300 APs at 10 Hz) was employed to mobilize a larger SV pool. As previously, the peak amplitude was unaltered between genotypes, yet importantly, the post-stimulus decay traces revealed a significantly slower time course of endocytosis in mutant cells versus *Tbc1d24^WT^* controls (Fig. 4B, C). We also investigated during-stimulus endocytosis using the drug folimycin to block SV re-acidification (32). Cells were stimulated initially at 10 Hz for 30 s without folimycin, allowed to rest, followed by a further 120 s at 10 Hz in the presence of the drug (Fig. 4D). As expected, in *Tbc1d24^WT^* neurons, the folimycin treatment increased the response to a 30 s stimulus compared to untreated cells as a measure of active, during-stimulus SV recycling (Fig. 4D). Strikingly, this increase in the response was almost absent in *Tbc1d24^tm1b^* neurons, suggesting a significant recycling deficit during stimulation (Fig. 4D). To further quantify the time course of net endocytosis during stimulation, the difference between the data acquired in the absence or presence of folimycin was calculated, revealing a strong (>3-fold) reduction in endocytic rate in *Tbc1d24^tm1b^* compared to *Tbc1d24^WT^* cells (Fig. 4E). Calculating the magnitude of endocytosis during the 300 AP protocol revealed a significant 4- to 5-fold reduction in mutant neurons compared to controls (Fig. 4F).

To address whether these functional defects caused by TBC1D24 haploinsufficiency were accompanied by modification in SV distribution at the synapses, we studied the synaptic ultrastructure by transmission electron microscopy (TEM) (Fig. 5A). Conventional TEM analysis of nerve terminals from *Tbc1d24^tm1b^* mice revealed a preserved density of total as well as docked SVs with respect to *Tbc1d24^WT^* neurons (Fig. 5B, C), whereas a significant increase in the density of endosomal-like structures was observed in mutants (Fig. 5D). To investigate this further, three-dimensional reconstructions of nerve terminals were carried out (Fig. 5E). Interestingly, these data suggested that while the number of SVs, docked SVs and endosome-like structures in *Tbc1d24^tm1b^* were unaffected (Fig. 5F–H), a striking 3-fold increase in the endosomal volume was observed (Fig. 5I). In summary, our data from the *Tbc1d24^tm1b^* model of TBC1D24 haploinsufficiency indicate an important function for the gene in neuronal development and survival alongside a critical role in presynaptic SV recycling in mammalian neurons.

## Discussion

Here we have established the essential role of TBC1D24 at the mammalian synapse, revealing that genetic conditions mirroring haploinsufficiency in TBC1D24 patients lead to defects in neuronal development, spontaneous neurotransmission and SV recycling. We began by surveying disease mutations throughout the TBC1D24 protein and demonstrated that all were likely to act as loss-of-function alleles when the endogenous protein was absent. It is noteworthy that the TBC and TLDc domains have been associated with independent functions, including lipid membrane association (16) and oxidative stress resistance (20), respectively. Crucially, our new data reveal that the function of both domains must be considered in concert with respect to neuronal development and survival, demonstrating the complexities of genotype–phenotype correlations in TBC1D24-associated diseases. Indeed, there are several clinical examples of biallelic *TBC1D24* mutations—one in each domain—where the disease phenotype is more severe than those that are monoallelic (12). Thus, the availability of a three-dimensional structure of the entire human protein will facilitate future predictions regarding potential allosteric interactions between the domains, as well as the phenotypic outcomes of specific pathogenic variants. For example, our discovery that disease-causing mutations in both the TBC and TLDc domains can influence cellular survival is pertinent to the neurodegeneration observed in a subset of TBC1D24 patients (12,13,33), in addition to a proposed role for oxidative stress in epilepsy (34).

Our new insights into TBC1D24 function at the mammalian synapse share some important parallels with previous work on this protein and its orthologues, although some key differences are apparent that likely reflect the experimental approaches used in the earlier studies. Acute knockdown of rat *Tbc1d24* results in cortical migration deficits (17), although the lack of a similar phenotype in *Tbc1d24^tm1b^* mice after constitutive genetic disruption *in vivo* may be the result of functional compensation during early neurodevelopment. Acute *Tbc1d24* knockdown also resulted in decreased spontaneous neurotransmission but alongside a reduction in glutamatergic synapse density (17); yet, these data must be considered in the context of a surrounding population of WT cells unlike the genetic model we present here. In loss-of-function models of the *Drosophila* orthologue *sky*, initial studies revealed an increased RRP of SVs at the NMJ with a concomitant increase in spontaneous activity (14); both are ultrastructural and physiological features that differ from our *Tbc1d24^tm1b^* model. Functionally, it was proposed that *sky* acts as a GTPase-activating protein for Rab35 and this underlies the endosomal-mediated quality control of SV proteins; currently, it is unknown whether this mechanism is conserved at a mammalian synapse with a larger complement of synaptic regulatory proteins. Interestingly, TBC1D24 was proposed to regulate the activation state of ADP-ribosylation factor 6 (ARF6) (17,18), and the slowed kinetics of SV recycling observed in *Tbc1d24^tm1b^* neurons is reminiscent of acute *Arf6* silencing (32). Thus, TBC1D24 could act as a regulatory hub for GTPases, which in turn differentially drives SV trafficking dependent on the cellular context; indeed, the common identification of an enlarged endosomal-like compartment in both *Tbc1d24^tm1b^* and *sky* mutants further supports a vital role for TBC1D24 in the dynamic regulation of SV recycling. Moreover, ARF6-dependent trafficking has been recently implicated in inner-ear hair cell function, suggesting that membrane trafficking defects in hair cells can be implicated in TBC1D24-associated deafness (35).

Our new findings define the significance of TBC1D24 for mammalian presynaptic function, and reveal that even partial loss-of-function mutations are likely to influence fundamental aspects of SV trafficking. These data place TBC1D24 alongside other more established synaptic proteins that are mutated in genetic forms of epilepsy but also play key roles in pathways essential for neurotransmission and synaptic homeostasis; for example, loss-of-function mutations in STXBP1 and SYN1 cause seizures and neurodevelopmental delay including autism in some SYN1 cases (4,6,36), and mutations in STX1B are associated with febrile epilepsy syndrome and myoclonic epilepsy (7,8). Furthermore, proteins more recently defined as being important for synaptic function can also result in pleiotropic clinical effects, such as proline-rich transmembrane protein 2 (PRRT2), where multiple mutations are associated with a range of disorders that include familial infantile seizures, paroxysmal kinesigenic dyskinesia and hemiplegic migraine (37). More specifically, we show here that TBC1D24 possesses new functional similarities to proteins such as activating protein 2 (AP-2) that are essential for maintaining endosomal dynamics in the nervous system (38). Despite these well-studied examples, there is still much to learn regarding the molecular mechanisms that underlie the function of presynaptic proteins in disease, particularly when a spectrum of disease type and severity is caused by mutations in the same gene. Our new data therefore help to establish common synaptic mechanisms that could underlie the varied effects of *TBC1D24* mutations in disease.

## Materials and Methods

### Animals

Mice were housed with *ad libitum* access to food and water on a 12:12 light–dark cycle. *Tbc1d24^tm1a(EUCOMM)Hmgu^* mice were generated as part of the large-scale mouse knockout programme (39) with loxp sites flanking exons 3–5 of the gene (Ensemble reference ENSMUST00000097376.9). *Tbc1d24*^*tm1a(EUCOMM*)*Hmgu*^ animals were crossed with mice expressing a ubiquitous cre-recombinase (C57BL6/J background) to generate *Tbc1d24^tm1b^* mutants in which the first 380 amino acids of the Tbc1d24 protein sequence are deleted from the mutant allele in all tissues. Complete cre-mediated generation of the *tm1b* allele was confirmed by genotyping from genomic DNA samples from ear clips and confirmed in brain tissue. *Tbc1d24^tm1b^* mice were backcrossed to C57BL6/J for a further six generations. All experiments were conducted from mice carrying a single *Tbc1d24^tm1b^* allele crossed to WT C57BL/6 J animals to generate the experimental cohorts. Experimenters were blinded to the genotypes for all experiments. All experiments were carried out under the UK (Home Office) regulations (Home Office Licence PPL 30/3353 held by Peter Oliver) and the guidelines established by the European Communities Council (Directive 2010/63/EU of March 4th, 2014) approved by the Italian Ministry of Health (authorization n. 73/2014-PR and n. 1276/2015-PR).

### Antibodies and reagents

The antibodies used are as follows: Actin (Abcam ab8226), Gapdh (Biolegend 919501 and Covance MMS580S), HA (Sigma H6908 and Roche 11867423001), Tbc1d24 (Abcam ab101933 and Aviva ARP57438), MYC (Sigma M4439), Map2 (Millipore AB5622), Tau (Millipore MAB3420), NF200 (Sigma N4142), vGLUT1 (Synaptic System 135303), Homer1 (Synaptic System 160011), NeuN (Millipore ABN91), Ctip2 (Abcam ab18465), Cux1 (Abcam ab140042), cleaved caspase-3 (Cell Signaling Technology 9661) and Tbr1 (Abcam ab31946) and anti-mouse, anti-rat or anti-rabbit Alexa 594 or Alexa 488 for secondary antibodies (Life Technologies). All reagents for cell culture were from Gibco, unless otherwise specified.

### Constructs and mutagenesis

Human TBC1D24 and the associated disease or experimental mutations were expressed from a pcDNA3.1 vector (Invitrogen) as previously described (13).

### Cell culture and neurite growth and cell death assay

Neuro-2a (N2a) cell lines were maintained as a monolayer in DMEM medium containing glutamax supplemented with 10% fetal calf serum (FCS), 100 unit/ml penicillin and 100 μg/ml streptomycin at 37°C in a humidified atmosphere of 5% CO_2_. N2a cells were plated on coverslips pre-treated with poly-ornithine. Cells were transfected the following day using Fugene 6 (Promega) as per manufacturer’s protocol. For neurite growth assay, culture medium was replaced with FCS-free culture medium before transfection for 48 h to induce neurite differentiation and growth. Neurite length was measured using ImageJ software (National Institutes of Health). For cell death assay, after 48 h transfection, culture medium was replaced with fresh complete medium containing either vehicle or arsenite (Sigma, 250 μm) for 5 h. Pyknotic nuclei were visualized by DAPI staining. Percentage of cell death was calculated as the number of pyknotic nuclei over the total number of nuclei in one field of view.

### Cortical and hippocampal cell culture

Cortices and hippocampi from *Tbc1d24^WT^* and *Tbc1d24^tm1b^* postnatal day 1 (P1) mouse pups were dissected and meninges removed in cold Hanks' Balanced Salt solution. Cortices were incubated in trypsin for 15 min at 37°C, followed by 5 min of incubation at room temperature with trypsin inhibitor (Life Technologies), and triturated to dissociate cells. Cortical neurons were plated on poly-ornithine pre-coated glass coverslips in culture medium (Neurobasal-A, 2% FCS, 2% B27, 1% L-glutamine). Hippocampi were incubated in diluted trypsin (1:50) in HBSS for 10 min at room temperature before 1 ml of culture medium (Neurobasal-A, 2% FCS, 2% B27, 1% L-glutamine) was added and hippocampi were triturated. Hippocampal neurons were plated on coverslips pre-coated with PDL (Sigma) and Fibronectin (Sigma). Half the culture medium was changed every other day. Neurons were cultured *in vitro* for 5 days before assessing neurite outgrowth or cell death after treatment with vehicle or arsenite (Sigma, 150 μm for 4 h).

### Western blotting

For protein extracts, brains or cells were homogenized in RIPA buffer (150 mM NaCl, 1% Triton X-100, 0.5% sodium deoxycholate, 0.1% SDS, 50 mM Tris pH 7.5, all Sigma) complemented with protease inhibitor cocktail (Cell signalling) with ceramic beads (Precellys; for tissue). Extracts were incubated for 30 min on ice and centrifuged at maximum speed for 30 min at 4°C to clear protein extracts. Protein concentration was quantified using a Pierce BCA assay (ThermoScientific). Proteins were run on pre-cast NuPAGE Bis-Tris gels (Invitrogen) and transferred as per the manufacturer’s protocol.

### *In vitro* immunocytochemistry

Neurons were washed once in PBS and fixed with 4% paraformaldehyde (PFA) for 15 min at room temperature. Cells were blocked with blocking buffer [5% goat serum (Vector Labs), 0.5% triton X-100] in PBS for 1 h at room temperature and incubated with primary antibodies overnight at 4°C, followed by incubation with secondary antibodies for 2 h at room temperature. Staining was visualized using a fluorescence microscope (Leica).

### Generation of Tbc1d24^Δ/Δ^ N2A cell lines

Cells were generated using a CRISPR-Cas9 strategy: single guide RNAs (sgRNAs) target sites were selected within the mouse Tbc1d24 coding sequence using the CRISPR design tool (crispr.mit.edu) to minimize potential off-targets. sgRNA sequences were cloned into the puromycin cassette-containing pX459 vector (Addgene #62988, a kind gift of Feng Zhang) as annealed pairs of 24 nt oligos into the *Bbs* I site, and tested for efficacy by transfection into N2A neuroblastoma cells using Fugene 6 (Promega) followed by high-resolution melt analysis (HRMA) (40). An sgRNA target site in a splice donor site (AGCATGGGTACAGCCTAAGCAGG: sgRNA #742) was chosen based on these data for generating clonal knockout N2A lines. Two additional sgRNA, targeting either mid exon 4 or 5, respectively, were tested to control for any off-target effects: sgRNA #743 (TGGCAGAGGGCAAAGCGCTCAGG) and sgRNA #744 (TTTGAGTGGTCTTGATGAGCAGG). N2A cells in a 1.9cm^2^ well were transfected for 36 h with Fugene 6 (Promega) followed by treatment with 2.5 μg/ml puromycin for 48 h. The surviving cells were plated in 63cm^2^ dishes to generate single cell clones. Cells were grown 5–7 days until the appearance of colonies. Individual colonies were trypsinized and reseeded to 1.9cm^2^ wells. Clones were screened using HRMA with primers (F: AGACATTTGGTCCTGGATCC and R: CAGCTACCCTATGATTTCCTG) using Hotshot Diamond PCR mix (Clent Lifescience) with LCGreen plus dye (BioFire Diagnostics) on a Light Scanner (Idaho Technology Inc). Three independent clones were characterized phenotypically for sgRNA #742, and one clone was characterized for sgRNA #743 and sgRNA #744, with similar results. Results for sgRNA #742 are presented only. The positive clones were tested for loss of Tbc1d24 protein expression by western blotting. The identity of the genomic indels was determined by cloning the critical region into a TA-cloning vector (pcDNA4, Invitrogen) and sequencing 40 clones from each independent cell line. No WT sequences were identified in any sample.

### Modelling

Models for TLDc and TBC domains were individually built using Modeller, provided sequence alignments produced with Clustal Omega (41). The TBC domain was modelled using PDB 2Q88 as template (22.93% sequence identity), while the TLDc domain was built using PDB T4ACJ (34.76% sequence identity). For the latter domain, sequence alignment indicates that a 47-amino acid-long insertion is present between residue K448 and E495. Secondary structure prediction by JPRED (42) indicates that the region is dominated by random coil, besides a possible alpha helix between residues P481 and A485 (i.e. 89.4% random coil). This region was therefore omitted from the model, given the high uncertainty about its location. Both models were independently relaxed via molecular dynamics simulations using the Amber ff14 force field (43), and the NAMD molecular dynamics engine (44). Structures were solvated in a box of TIP3P water, and the simulation boxes were neutralized by adding Na+ counterions. In the case of the TLDc domain, the unfolded K448-E495 region was not included. The resulting systems were energy-minimized via 2000 conjugate gradient steps, and equilibrated for 1 ns in the nVT ensemble, at a pressure of 1 atm and temperature of 300 K. A 2 fs time step was adopted, with all covalent bonds controlled by SHAKE, a distance cutoff at 12 Å and Particle Mesh Ewald to treat long-range electrostatic interactions. Langevin dynamics were exploited to control temperature and pressure, with a 0.1 ps^−1^ damping. Both systems were then simulated for 50 ns in the nPT ensemble, and their root-mean-square deviation (RMSD) and secondary structure assessed against the initial structure every 0.1 ns. To analyze the evolution of secondary structure, each simulation frame was submitted to the DSSP software (45), and its output used to determine the quantity of residues in structured regions. We considered as structured all amino acids in an α-helix, β-bridge, extended strand, 3–10 helix and π-helix conformation. We thus derived the percentage of amino acids maintaining a structured conformation on each simulation frame, as compared against the initial structure. To characterize the neighborhood relationships of disease-related amino acids, we calculated the pairwise distance between their α-carbons on the last structures of both TBC and TLDc domains’ simulations. These distances were then submitted to a Unweighted Pair Group Method with Arithmetic Mean hierarchical clustering algorithm as implemented in Python’s SciPy package. Image rendering was performed with VMD (46).

### Scanning electron microscopy

Inner ears were collected and processed for SEM, as previously described (47). Briefly, mice were culled by cervical dislocation and inner ears were removed and fixed in 2.5% glutaraldehyde in 0.1 M phosphate buffer. Following decalcification in 4.3% EDTA, cochleae were sub-dissected to expose the sensory epithelia and subjected to processing with an alternate incubation in 1% osmium tetroxide and 1% thiocarbohydrazide, before dehydration in increasing concentrations of ethanol. Samples were critical point dried using an Emitech K850 (EM Technologies Ltd), mounted on stubs using silver paint (Agar Scientific) and sputter coated with platinum using a Quorum Q150R S (Quorum Technologies). Cochleae were examined using a JEOL JSM-6010LV scanning electron microscope. Hair cell stereocilia bundle counts were performed by counting the number of outer hair cell and inner hair cell bundles adjacent to ten pillar cells in the apical (<180° from apex), mid (180–450° from apex) and basal (> 450° from apex) regions of the cochlea. At least six ears (one ear per mouse) were analysed for each genotype.

### Auditory brainstem response

ABR testing was performed to investigate auditory function, as described previously (47). Briefly, mice were anaesthetized with a mixture of ketamine and xylazine and placed on a heated mat inside a sound-attenuated chamber. Electrodes were placed subdermally over the vertex (active), right mastoid (reference) and left mastoid (ground). Broadband click and frequency-specific tone-burst stimuli (8 kHz, 16 kHz and 32 kHz) were presented free-field to the right ear of the mouse, starting at 90 decibel sound pressure level (dB SPL) and decreasing in 5 dB increments. ABR responses were collected, amplified and averaged using TDT System 3 hardware (Tucker Davies Technology) and BioSigRZ software. Auditory thresholds were defined as the lowest dB SPL at which an ABR trace pattern could be clearly distinguished.

### Behavioural testing

As a measure of righting behaviour and related motor function, mice at P6 were placed on their back with their limbs pointing upwards and the time needed to turn over and restore their normal prone positions was recorded and averaged over three consecutive trials. As a measure of postural stability and mobility test, or negative geotaxis, mice at P7 were placed on a grid, tilted 45° to the plane, with their head facing downwards. Animals that could rotate a full 180°, face up and could climb the grid within a maximum time of 30 s were considered to have fully acquired this reflex. To measure early development of locomotion, mature walking behaviour was measured at P16 when the animal was able to move its body completely supported by the four limbs without dragging the belly over the surface over a 60 s trial. To measure novelty-induced locomotor activity (LMA), experiments were conducted in the light phase between 1000 and 1100 h. Ambulations were measured in transparent plexiglass cages (20 × 35 cm), equipped with infrared photobeams (San Diego Instruments) over a total of 1 h. The central area was defined as a region 10 × 15 cm in the arena.

### *In situ* hybridization

A digoxigenin-labelled riboprobe was synthesized from linearized plasmid pCR4-TOPO (Invitrogen; nucleotides 2705–3558 of NM_001163847). Tissue samples were snap frozen in Optimal Cutting Temperature and 15 μm sections were cut using a cryostat (Leica) and mounted onto Superfrost Plus slides (Avantor). Probe hybridization, washing and signal detection using an alkaline phosphatase-conjugated anti-DIG antibody was carried out as previously described (20).

### Neonatal neuropathology

Mice were perfused transcardially with 0.1 M PBS, followed by 4% PFA. Brains were dissected out and post-fixed in 4% PFA at 4°C overnight, then transferred to 30% sucrose for cryoprotection. Brains were cut at 15 μm using a cryostat (Bright). For Nissl staining, sections at 60 μm intervals representing equivalent regions of the somatosensory or motor cortex were quantified. For cortical layer and hippocampal immunostaining of *Tbc1d24^WT^* and *Tbc1d24^tm1b^*, five sections representing equivalent regions of the somatosensory or motor cortex at 60 μm intervals were mounted on slides and blocked in 5% donkey or goat serum in PBS with 0.3% Triton X-100. Primary antibodies were incubated in the same blocking solution overnight at 4°C. Immunostaining for Tbr1 and Ctip2 required additional retrieval in 10 mM sodium citrate (Sigma) pH 6.0 buffer for 20 min prior to blocking. The appropriate secondary antibodies were applied for 2 h at room temperature. Quantification of immunopositive cells was carried out from a fixed field of view using an ImageJ plug-in using a Leica microscope.

### pHluorin dye analysis

Hippocampal neurons were transfected with 3 μg 2x SypH DNA with Lipofectamine 2000 (Invitrogen) for 1 h before the medium was replaced and neurons cultured for further 7 days before assaying (48). Coverslips were mounted in a Chamlide stimulation chamber (Live Cell Instrument, Seoul, Korea) on the stage of an Olympus IX-71 inverted microscope fitted with a 100 X, NA 1.40 UPlanSApo objective and an Andor iXon EM CCD camera. Fluorescence illumination was supplied by a 100 W mercury lamp used with appropriate neutral density filters and shuttered (Uniblitz) during all non-data acquisition periods. A 460–495 nm excitation/510–550 nm emission filter set was used for pHluorins. APs were evoked by passing 40 V, 1 ms current pulses from a custom-made stimulation box via platinum electrodes. Stimulation, image acquisition and shuttering were all under the co-ordinated control of WinWCP software. Imaging was carried out at room temperature in a Tyrode’s buffer containing (in mM): 119 NaCl, 2.5 KCl, 2 CaCl_2_, 2 MgCl_2_, 25 HEPES (pH 7.4) and 30 glucose. 10 μM NBQX and 50 μM APV were added to block recurrent activity. Where used, folimycin was added to a final bath concentration of 8 nM. Images were analysed in ImageJ using the Time Series Analyzer plugin. Visible varicosities were selected for analysis with a 2 μm region of interest. Data exported from ImageJ were background adjusted and normalized to the mean baseline pre-stimulus signal in order to adjust for differing pHluorin expression levels. Peak fluorescence in all experiments was taken at the end of the stimulation period. Analysis was performed in Microsoft Excel using custom-written macros.

### TEM of primary neurons

Cultured cortical neurons derived from Tbc1d24^WT^ and Tbc1d24^Tm1b^ mice were fixed at 14–18 DIV with 1.2% glutaraldehyde in 66 mM sodium cacodylate buffer, post-fixed in 1% OsO4, 1.5% K4Fe(CN)6, 0.1 M sodium cacodylate, en bloc stained with 1% uranyl acetate, dehydrated and flat-embedded in epoxy resin (Epon 812, TAAB). After baking for 48 h, the glass coverslips were removed from the Epon block by thermal shock and neurons were identified by means of a stereomicroscope. Embedded neurons were excised from the block, and mounted on a cured Epon block for sectioning using an EM UC6 ultramicrotome (Leica Microsystems). Ultrathin sections (60–70 nm thick) were collected on 200-mesh copper grids (Electron Microscopy Sciences) and observed with a JEM-1011 electron microscope (Jeol) operating at 100 kV using an ORIUS SC1000 CCD camera (Gatan, Pleasanton). For each experimental condition, at least 30 images of synapses were acquired at 10 000× magnification (sampled area per experimental condition: 36 μm^2^). Synaptic profile area, SV number and distribution relative to the active zone (AZ) were determined using ImageJ software. For the 3D reconstruction, the standard TEM sample preparation protocol was followed, and samples were embedded in Epon resin. Serial 60-nm-thick sections were collected on carbon-coated copper slot formvar and carbon-coated grids, and serial synaptic profiles acquired. Serial sections were aligned using Midas from IMOD software. Synapses with one single AZ, at least one docked SV and greater than 100 SVs were reconstructed with IMOD software.

### Electrophysiological recordings

Hippocampal neurons were recorded at 13–15 DIV. Patch pipettes, prepared from thin borosilicate glass, were pulled and fire-polished to a final resistance of 4–5 MΩ when filled with standard internal solution. Current-clamp recordings were performed at a holding potential of −70 mV, and AP firing was induced by injecting current steps of 10 pA lasting 500 ms. Voltage-clamp recordings for spontaneous miniature post-synaptic currents (mPSCs) were acquired at 10–20 kHz sample frequency at the same holding potential. All experiments were performed at room temperature. Data acquisition was performed using PatchMaster program (HEKA Elektronic). For all experiments, cells were maintained in extracellular standard solution (Tyrode) containing (in mM): 140 NaCl, 2 CaCl_2_, 1 MgCl_2_, 4 KCl, 10 glucose, and 10 HEPES (pH 7.3 with NaOH). All reagents were bought from Tocris, unless otherwise stated. For the analysis of neuronal excitability, recordings in current-clamp configuration were performed in Tyrode extracellular solution in which D-(−)-2-amino-5-phosphonopentanoic acid (D-AP5; 50 μM), 6-cyano-7 nitroquinoxaline-2,3-dione (CNQX; 10 μM), bicuculline methiodide (30 μM) and (2S)-3-[[(1S)-1-(3,4-dichlorophenyl)ethyl]amino-2-hydroxypropyl] (phenylmethyl)phosphinic acid hydrochloride (CGP58845; 5 μM) were added to block NMDA, non-NMDA, GABA-A and GABA-B receptors, respectively. The internal solution (K-gluconate) was composed of (in mM) 126 K gluconate, 4 NaCl, 1 MgSO_4_, 0.02 CaCl_2_, 0.1 BAPTA, 15 glucose, 5 HEPES, 3 ATP and 0.1 GTP (pH 7.3 with KOH). Spontaneous post-synaptic currents (PSCs) were recorded in voltage-clamp configuration in the presence of TTX (300 nM) in the extracellular solution to block the generation and propagation of spontaneous APs. To isolate mEPSCs, bicuculline methiodide and CGP58845 were added to Tyrode extracellular solution. The amplitude and frequency of mEPSCs were calculated using a peak detector function using different appropriate threshold amplitude and area. The frequency, amplitude and kinetics of miniature EPSCs were analysed using the MiniAnalysis program and the Prism software (GraphPad Software, Inc.).

### Synapse density quantification

Hippocampal neurons were fixed at 13–15 DIV, in 4% formaldehyde and 4% sucrose in phosphate-buffered saline (PBS) for 5 min at room temperature. Cells were then washed three times in PBS, permeabilized and blocked for 30 mins in 5% FBS, 0.1% Triton X-100 in PBS. Primary antibodies diluted in 5% FBS in PBS were applied up to 2 h. Cultures were immunostained with vGLUT1 and Homer1, with Alexa-conjugated secondary antibodies and mounted using Prolong Diamond anti-fade reagent with DAPI (Invitrogen). 18–35 z-stack for each image (step size 0.35 μm) were acquired. Image analyses were performed with ImageJ superimposing stacks of each colour channel. The co-localization analysis was performed by evaluating the labelling of pre- and post-synaptic protein couples (vGLUT1/Homer1). Co-localization puncta with areas of 0.1–2 μm^2^ were considered *bona fide* synaptic boutons and their number per field automatically counted evaluated by using the ImageJ colocalization plug-in.

## Supplementary Material

Supplementary Data
