# Supplementary material for: The epilepsy-associated protein TBC1D24 is required for normal development, survival and vesicle trafficking in mammalian neurons
Source: Hum Mol Genet. 2018 Oct 17;28(4):584–97. doi: 10.1093/hmg/ddy370 (PMC6360273; doi:10.1093/hmg/ddy370)
Supplement: Supplementary Data [file ddy370_supp.zip › Finelli et al. suppl figure legends OCT.docx]

**Finelli MJ et al.**

**Supplementary Figure Legends**

**Supplementary Figure S1: No clustering of disease-associated mutations occurs in TBC1D24 from molecular dynamics simulation of the TBC domain. (A)** Evolution of root-mean-square deviation (RMSD) and secondary structure of molecular dynamics simulation of the TBC domain of TBC1D24; the TBC backbone RMSD converged at 3 Å after approximately 10 ns. **(B)** Plot reporting the percentage of amino acids maintaining a structured conformation, as compared against the first simulation frame; no significant folding or unfolding is observed, indicating that the domain model is equilibrated. **(C)** Dendrogram representations of the neighborhood relationship between disease-related amino acids in the TBC domain of TBC1D24. For each plot, the pairwise distance between α-carbons of selected amino acids was calculated and aggregated via a hierarchical clustering algorithm. In colour, clusters of amino acids at a distance less than 15 Å are indicated. No disease appears to be associated to mutation within a specific protein region. Note that for the TLDc domain, our structural models did not explicitly feature all the disease mutation sites.

**Supplementary Figure S2: Expression of TBC1D24 constructs.** Western blot of protein extracts from N2a cells transfected with the wild-type (WT) and mutant TBC1D24 constructs indicated. β-actin was used as a loading control.

**Supplementary Figure S3: *Tbc1d24^tm1b^* mice show no defects in early postnatal cortical or hippocampal development. (A-B)** Nissl staining at P15 indicating normal cortical and hippocampal structural organisation in *Tbc1d24^tm1b^* mice compared to controls. Cortical layers I-VI are shown as boxed region in (B). Scale bar: 0.5 mm (A) and 200 μm (B). **(C)** Nissl staining and cortical layer marker immunostaining using the antibodies shown from wild-type and littermate *Tbc1d24^tm1b^* cortical brain sections from the somatosensory cortex at P7; Scale bar: 200 μm. **(D)** Representative NeuN immunostaining of the CA1 field of the hippocampus at P7; Scale bar: 50 μm. Quantification of cell numbers **(E-I)** shows no significant differences between genotypes (*N* = 5 per genotype); unpaired *t*-test. Data are expressed as the mean ± SEM.

**Supplementary Figure S4: *Tbc1d24^tm1b^* mice show no abnormalities in early postnatal developmental milestones or activity. (A)** Weight gain over 8 weeks in wild-type and *Tbc1d24^tm1b^* mice. **(B)** Righting-reflex response at P6. **(C)** Percentage of animals showing normal negative geotaxis reflex at P7. **(D)** Percentage of animals showing a normal locomotor posture with trunk raised above the ground at P16. **(E-G)** Locomotor activity as quantified by beam breaks in a rectangular arena in P28 mice. The first 5 minutes of exposure to the area **(E)**, the following 55 minutes **(F),** and the percentage time spent in the central region of the arena **(G)** is shown. (*N* = 12 per sex of each genotype; data are combined for both sexes in panels B-D). No significant differences were identified. Data are expressed as the mean ± SEM.

**Supplementary Figure S5: *Tbc1d24^tm1b^* mice show no abnormalities in hearing response or inner ear hair cell pathology. (A)** Audio brainstem response (ABR) indicating no defects in hearing in *Tbc1d24^tm1b^* mice compared to littermate controls (*N* = 5-6 each genotype). **(B)** Electron micrographs of inner-ear hair cells at P16 demonstrating no defects in the number or morphology in *Tbc1d24^tm1b^* mice compared to littermate controls. Scale bars: 10 μm (left panels) 1 μm (right panels, boxed region as indicated). **(C)** Representative scanning electron microscopy of inner hair cell stereocilia from *Tbc1d24^tm1b^* and wild-type littermate control mice at P16; Scale bar = 1 μm. **(D-E)** Quantification of inner (IHC) and outer hair cell (OHC) stereocilia in the apical (<180° from apex), mid (180 – 450° from apex) and basal (> 450° from apex) regions of the cochlea at P16. (*N* = 5-6 of each genotype). Unpaired *t*-test with Welch’s correction. Data are expressed as the mean ± SEM.
