# Supplementary figures and images for: The epilepsy-associated protein TBC1D24 is required for normal development, survival and vesicle trafficking in mammalian neurons
Source: Hum Mol Genet. 2018 Oct 17;28(4):584–97. doi: 10.1093/hmg/ddy370 (PMC6360273; doi:10.1093/hmg/ddy370)

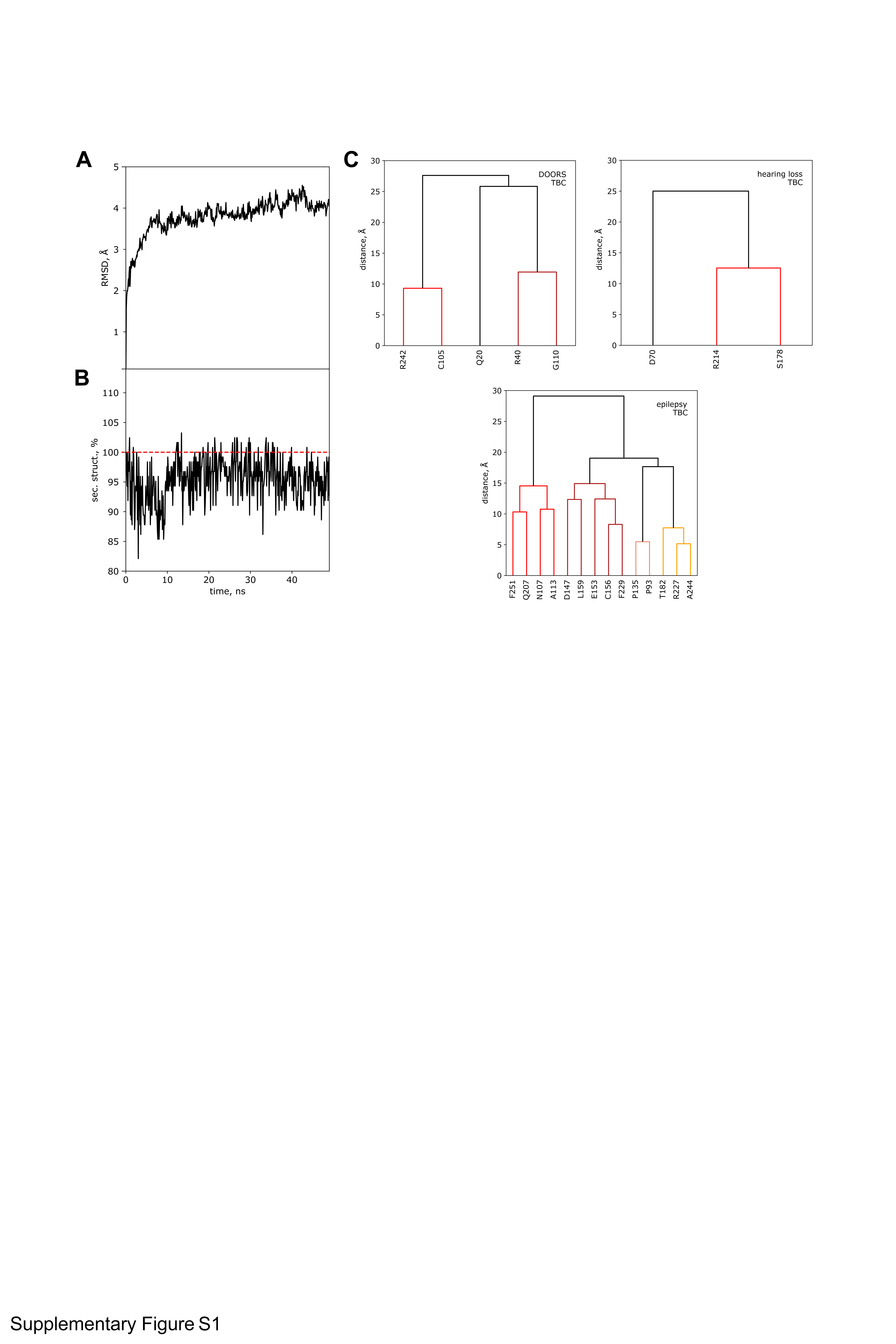

Supplement: Supplementary Data [file ddy370_supp.zip › Suppl Fig 1 OCT.tif]

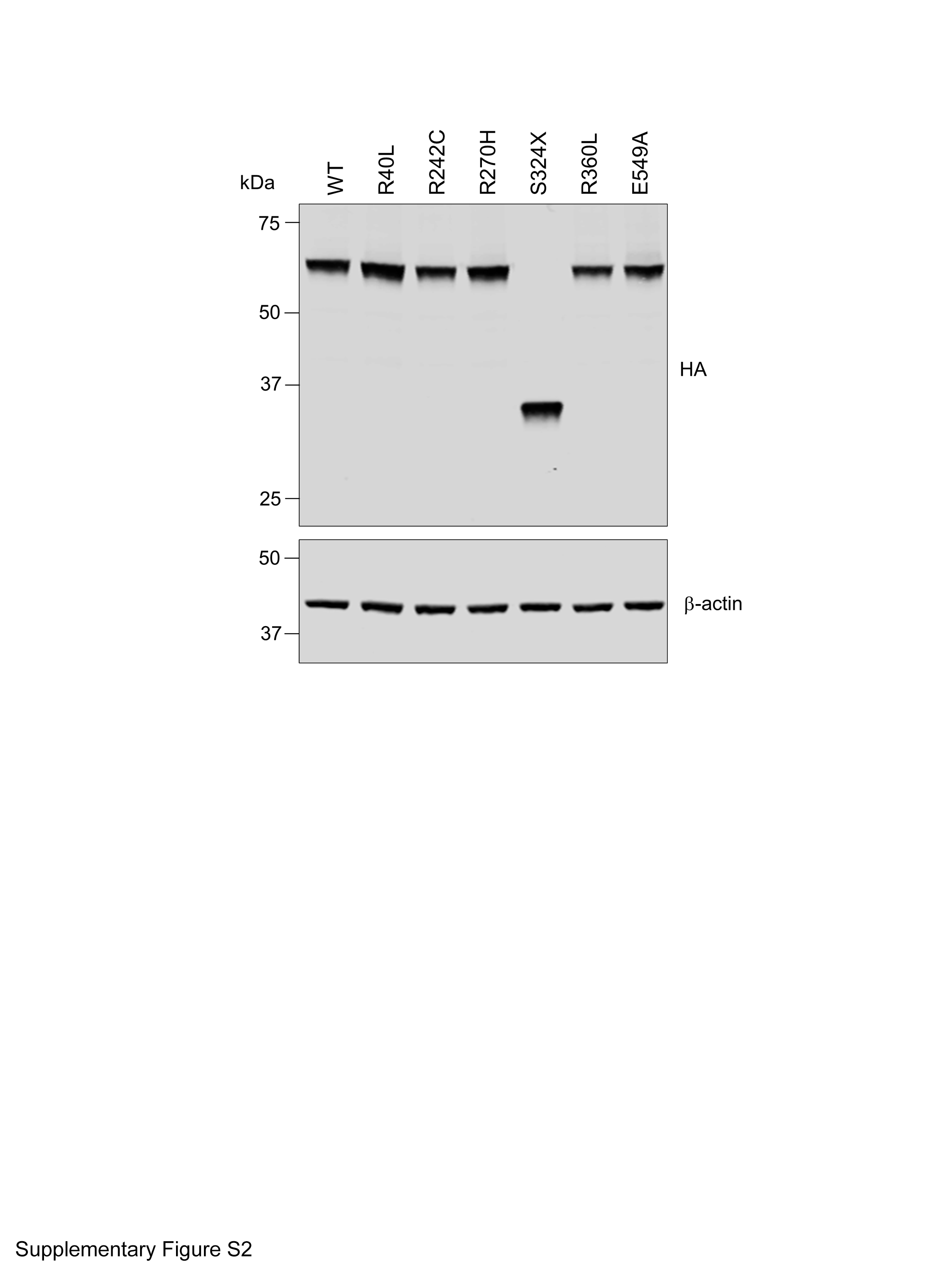

Supplement: Supplementary Data [file ddy370_supp.zip › Suppl Fig 2 OCT.tif]

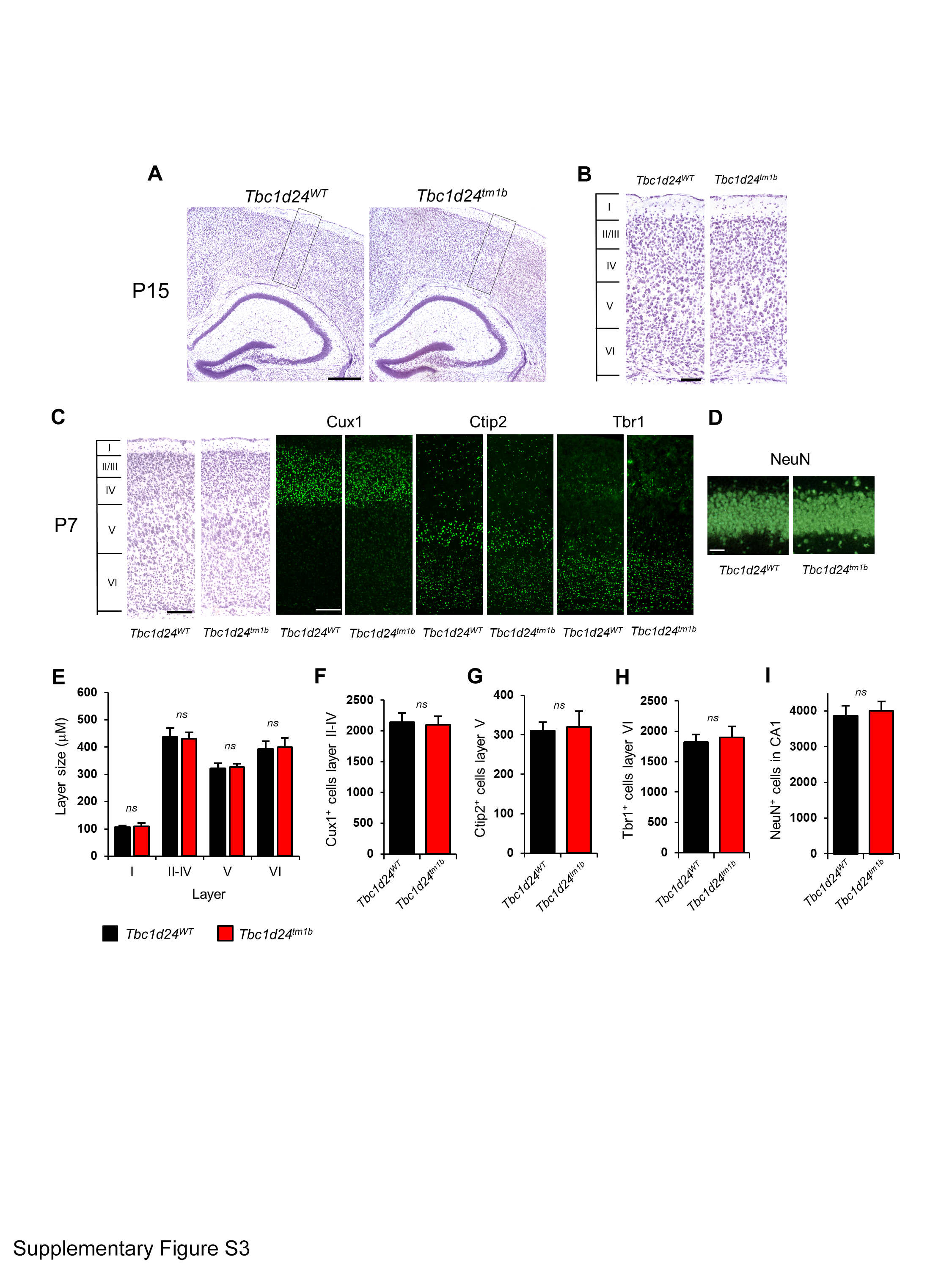

Supplement: Supplementary Data [file ddy370_supp.zip › Suppl Fig 3 OCT.tif]

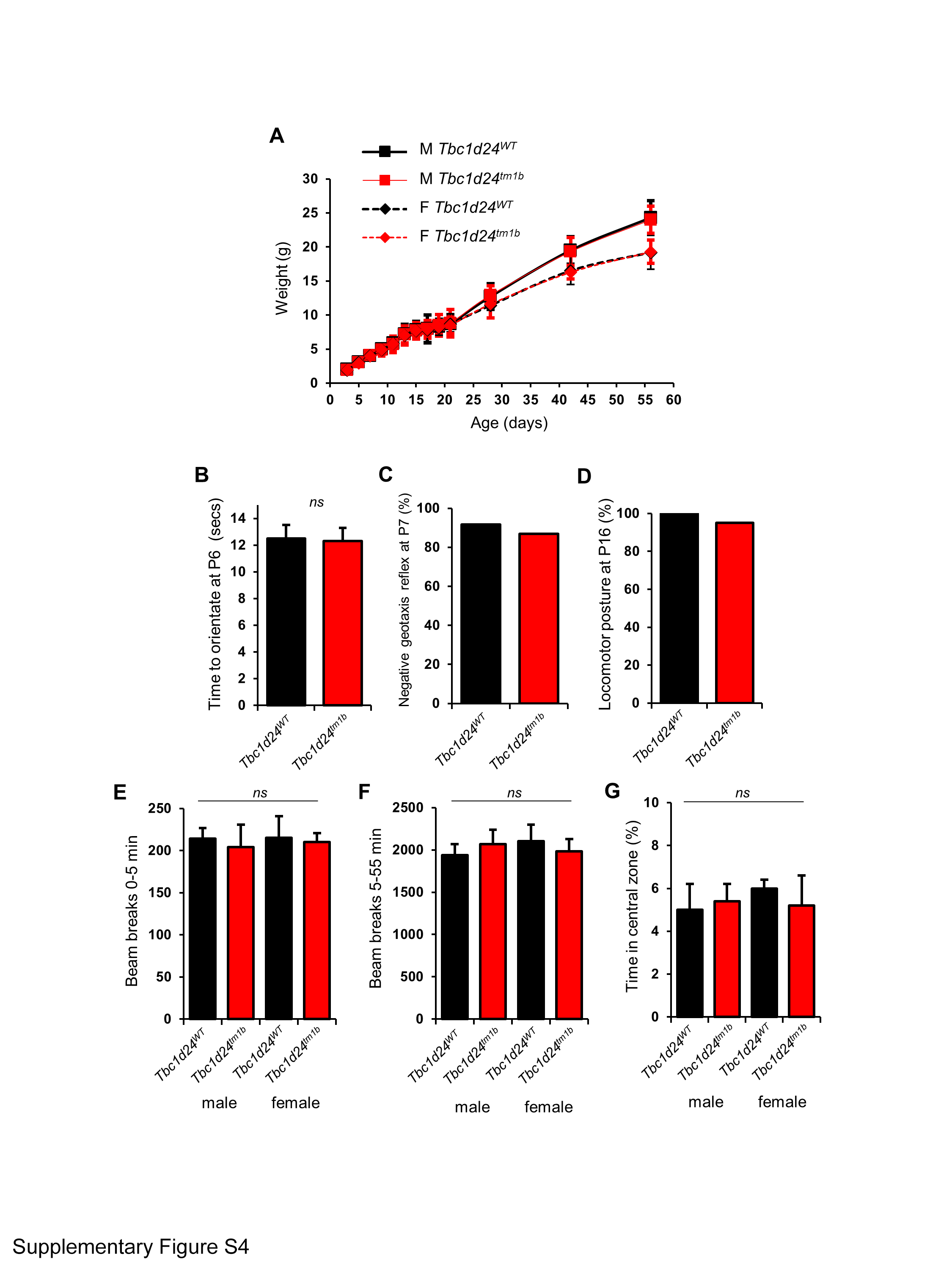

Supplement: Supplementary Data [file ddy370_supp.zip › Suppl Fig 4 OCT.tif]

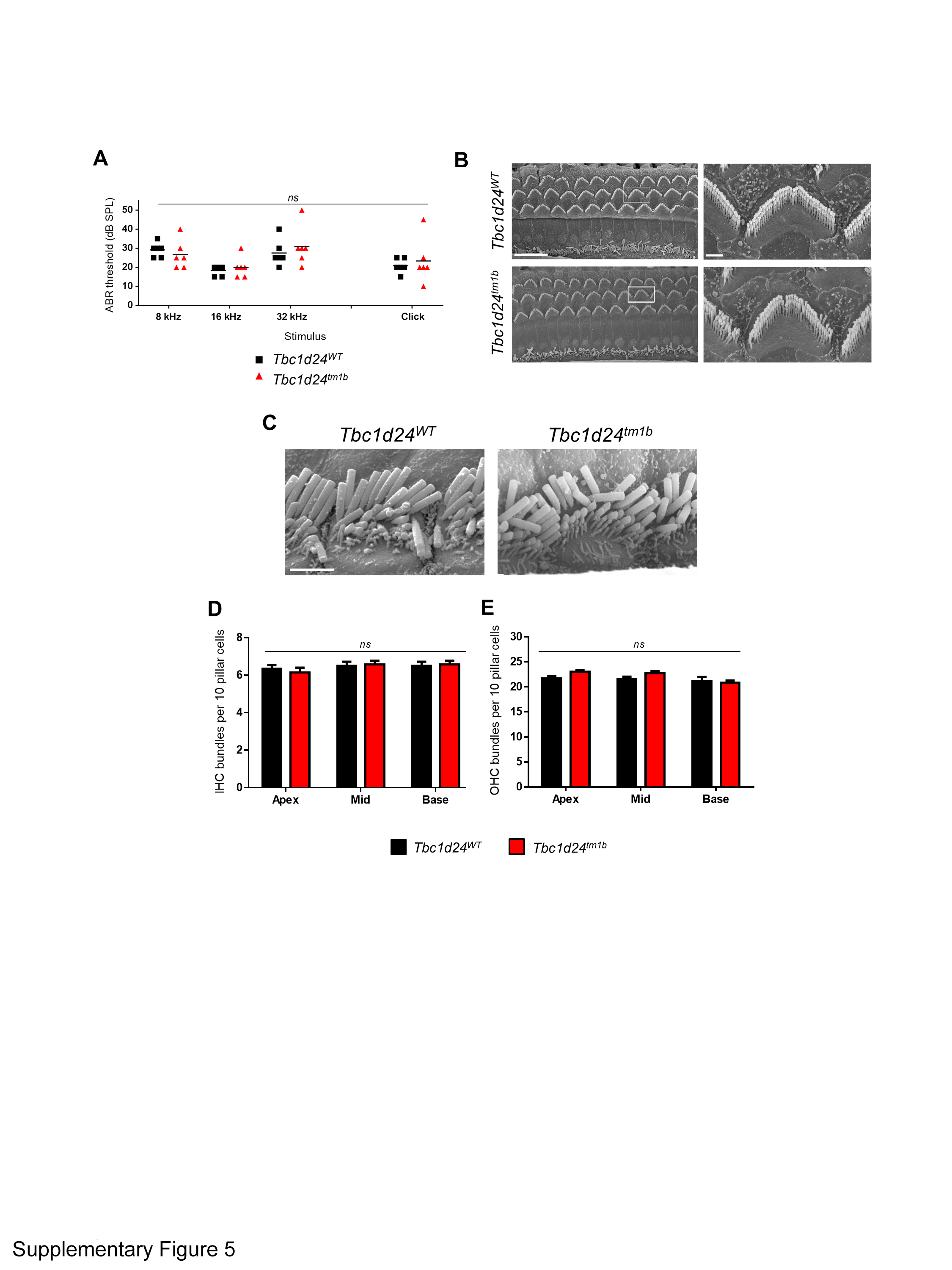

Supplement: Supplementary Data [file ddy370_supp.zip › Suppl Fig 5 OCT.tif]
